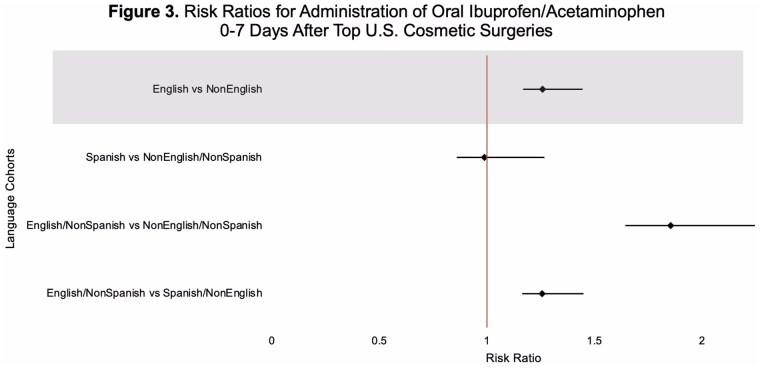# Opioid Prescription Disparities in Non-English Speaking Patients Following Most Common U.S. Aesthetic Surgeries

**DOI:** 10.1093/asjof/ojaf018.011

**Published:** 2025-05-13

**Authors:** Emily Long, Noelle Garbaccio, Audrey Mustoe, Jade Smith, Dorien Schonebaum, Lacey Foster, Morvarid Mehdizadeh, Justin Cordero, Bernard T Lee, Samuel J Lin

**Affiliations:** Beth Israel Deaconess Medical Center, Boston, MA; Beth Israel Deaconess Medical Center, Boston, MA; Beth Israel Deaconess Medical Center, Boston, MA; Beth Israel Deaconess Medical Center, Boston, MA; Amsterdam UMC, Vrije Universiteit, Amsterdam, Netherlands; Beth Israel Deaconess Medical Center, Boston, MA; Beth Israel Deaconess Medical Center, Boston, MA; Beth Israel Deaconess Medical Center, Boston, MA; Beth Israel Deaconess Medical Center, Boston, MA; Beth Israel Deaconess Medical Center- Harvard Medical School, Boston, MA

## Abstract

**Goals/Purpose:**

Post-operative pain management is a crucial aspect of recovery after aesthetic surgery. Effective pain control leads to quicker ambulation and return to work, and decreased rates of DVT/PE, chronic pain, and opioid dependence. Non-English-speaking patients frequently encounter difficulties communicating with the medical system, and previous studies in other surgical fields suggest discrepancies in post-operative pain management between English-speaking and non-English-speaking patients. In this study, we aimed to evaluate whether primary spoken language affects postoperative pain management prescriptions following aesthetic surgery.

**Methods/Technique:**

This study utilized TriNetX, a global health research platform that compiles data from electronic medical records in real-time. Ninety-seven healthcare organizations and 134,328,422 patients were queried for patients with a lifetime history of at least one of the United States’s top five aesthetic surgeries (liposuction, breast augmentation, abdominoplasty, mastopexy, or blepharoplasty), documented as SNOMED or CPT codes. TriNetX demographic codes for spoken language differentiated patients into two cohorts: English (En) and non-English (nonEn) speakers. Sub-cohorts were also broken down into (1) English, non-Spanish (En/nonSp); (2) Spanish, non-English (Sp/nonEn); and (3) non-English, non-Spanish (nonEn/nonSp) speakers. Propensity score matching was performed on thirteen characteristics that included patient age at first aesthetic surgery, sex, BMI, body weight, substance use, pain disorders, neoplasms, and socioeconomic and psychosocial circumstances. Risk analysis compared the proportions of patients prescribed intravenous opioids, oral opioids, and oral acetaminophen or ibuprofen 0-to-7 days after surgery across groups. Risk difference, risk ratio, and odds ratio were calculated for each pair of cohorts.

**Results/Complications:**

This study utilized TriNetX, a global health research platform that compiles data from electronic medical records in real-time. Ninety-seven healthcare organizations and 134,328,422 patients were queried for patients with a lifetime history of at least one of the United States’s top five aesthetic surgeries (liposuction, breast augmentation, abdominoplasty, mastopexy, or blepharoplasty), documented as SNOMED or CPT codes. TriNetX demographic codes for spoken language differentiated patients into two cohorts: English (En) and non-English (nonEn) speakers. Sub-cohorts were also broken down into (1) English, non-Spanish (En/nonSp); (2) Spanish, non-English (Sp/nonEn); and (3) non-English, non-Spanish (nonEn/nonSp) speakers. Propensity score matching was performed on thirteen characteristics that included patient age at first aesthetic surgery, sex, BMI, body weight, substance use, pain disorders, neoplasms, and socioeconomic and psychosocial circumstances. Risk analysis compared the proportions of patients prescribed intravenous opioids, oral opioids, and oral acetaminophen or ibuprofen 0-to-7 days after surgery across groups. Risk difference, risk ratio, and odds ratio were calculated for each pair of cohorts.

**Conclusion:**

English-speaking patients were significantly more likely than non-English speaking patients to receive opioid prescriptions following aesthetic surgery. Additionally, English-speaking patients were more likely to receive prescriptions for multimodal pain control regimens. Our data suggests there may be broad-scale language-based disparities and potential under-treatment of pain in non-English speaking aesthetic surgery patients. Plastic surgeons may aim to establish standardized postoperative pain-control protocols to reduce language-based discrepancies in postoperative pain management.

**Figure:**
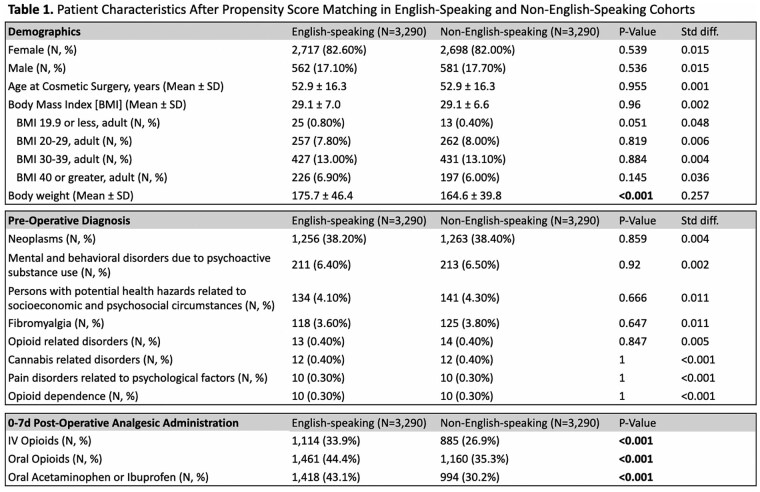

**Figure:**
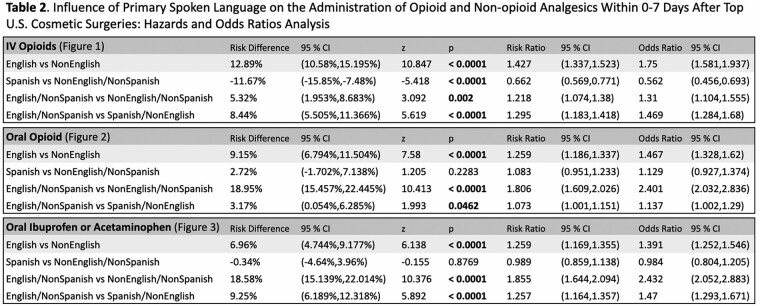

**Figure:**
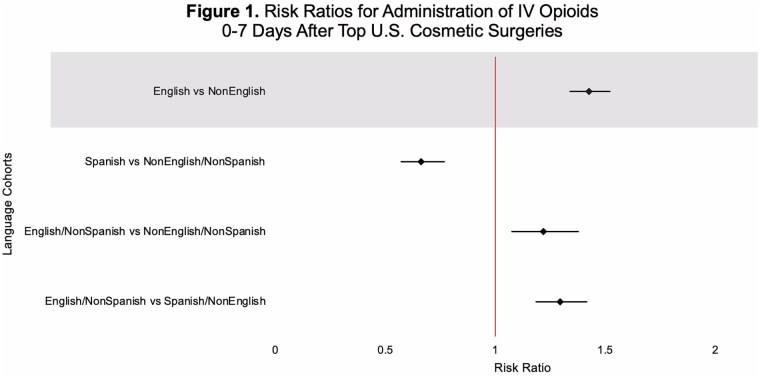

**Figure:**
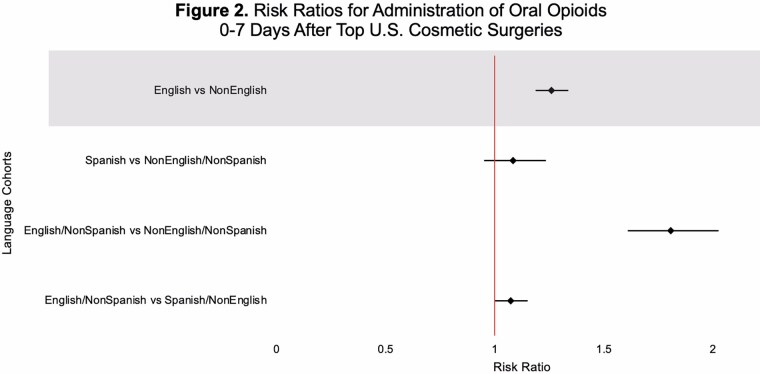

**Figure:**